# Supplementary material for: Strain-Dependent Impact of G and SH Deletions Provide New Insights for Live-Attenuated HMPV Vaccine Development
Source: Vaccines (Basel). 2019 Oct 30;7(4):164. doi: 10.3390/vaccines7040164 (PMC6963613; doi:10.3390/vaccines7040164)
Supplement: Supplementary file 1 [file vaccines-07-00164-s001.pdf]

Figure A1. HMPV-immunized mice showed significant differences in histopathology 5 days after viral challenge.

After intranasal immunization with $5x10^5$ $TCID_{50}$ of ΔSH-C-85473, ΔG-C-85473 or WT rC85473 virus, mice were inoculated with $1×10^6$ $TCID_{50}$ (LD_{50}) of rC-85473 3-weeks later. Tissues observations (x100 magnification) and measure of pulmonary edema scores were performed on mice lungs on day 5 post-challenge (n=2).

		pulmonary edema score
challenged mice mock-immunized	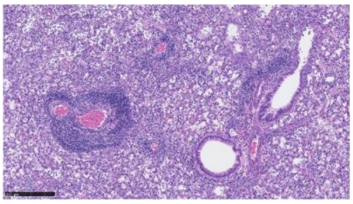	1.5
challenged mice rC-85473 WT immunized	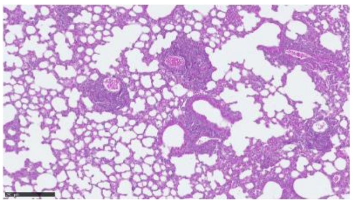	0
challenged mice ΔSH-C-85473 immunized	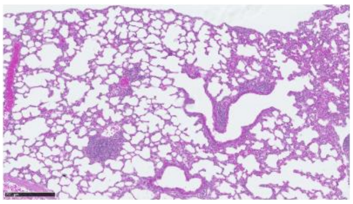	0
challenged mice ΔG-C-85473 immunized	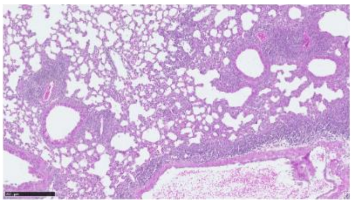	0
